# Supplementary material for: Protective Role of an Extract Waste Product from Citrus bergamia in an In Vitro Model of Neurodegeneration
Source: Plants (Basel). 2023 May 27;12(11):2126. doi: 10.3390/plants12112126 (PMC10255109; doi:10.3390/plants12112126)
Supplement: Supplementary file 1 [file plants-12-02126-s001.zip › plants-2403967-supplementary.pdf]

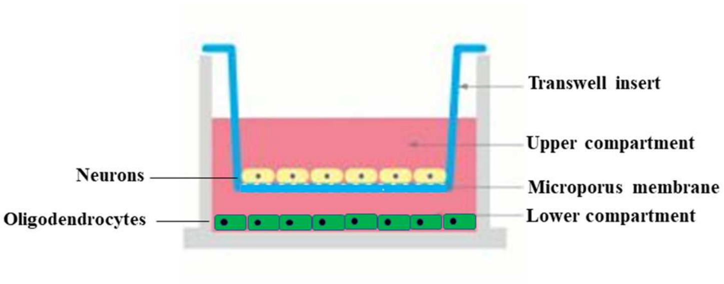

Supplementary Figure S1. . The model used in this experimental work

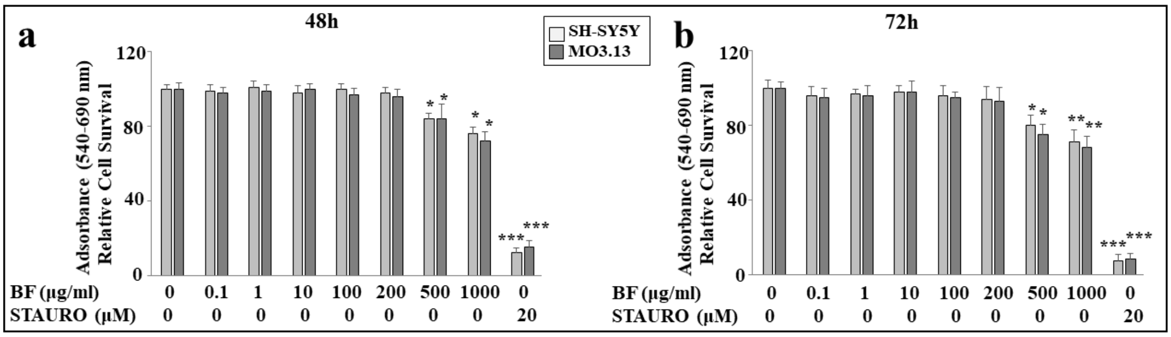

Supplementary Figure S2. Effects of BF on cell viability used for 48 and 72 h. In panel (**a**), the representation of cell viability following increasing concentrations of BF on neurons and oligodendrocytes for 48 h is shown. In panels (**b**), the representation of cell viability following increasing concentrations of BF on neurons and oligodendrocytes for 72h is shown. The experiments were conducted using MTT assay. Three independent experiments were carried out, and the values are expressed as the mean ± standard deviation (sd). * denotes $p < 0.05$ vs. the control; ** denotes $p < 0.01$ vs. the control; *** denotes $p < 0.001$ vs. the control. Analysis of variance (ANOVA) was followed by a Tukey–Kramer comparison test.
